# Physical activity and symptoms of depression and anxiety among professionals at a Brazilian university hospital: a cross-sectional study

**DOI:** 10.47626/1679-4435-2026-1520

**Published:** 2026-05-21

**Authors:** Luiz Paulo do Carmo Guanabens, Carla Zimerer, Lucas Lima Galvão, Anne Sulivan Lopes da Silva Reis, Rodrigo Luiz Vancini

**Affiliations:** 1 Universidade Federal do Espírito Santo, Educação Física, Vitória, ES, Brazil

**Keywords:** physical inactivity, mental health, occupational health.

## Abstract

**Introduction:**

Physical activity may influence symptoms of depression and anxiety, especially among healthcare professionals exposed to high levels of occupational stress.

**Objectives:**

To investigate the association between physical activity levels and symptoms of depression and anxiety in professionals at a Brazilian university hospital, using analyses based on continuous scores.

**Methods:**

This was a cross-sectional observational study conducted with 199 professionals from medical, healthcare, and administrative areas. Habitual physical activity, depression symptoms, and anxiety symptoms were assessed using standardized questionnaires. The association between physical activity and symptoms was analyzed using generalized linear models, adjusted for sex, age, and body mass index, considering both overall and area-specific analyses.

**Results:**

The sample was predominantly female, from the healthcare area, and sedentary. Regarding categorical prevalence, 44.2% of professionals presented symptoms of depression and 54.2% symptoms of anxiety. In analyses based on continuous scores, individuals classified as active and very active had lower depression and anxiety scores compared to sedentary individuals. Stratification by area of work confirmed the inverse association, especially among medical and healthcare professionals.

**Conclusions:**

Higher levels of physical activity were significantly associated with lower symptoms of depression and anxiety among professionals at a Brazilian university hospital.

## INTRODUCTION

Brazil has the largest public health care system in the world, the Brazilian Unified Health System (SUS), which ensures universal and free access to the population. Currently, SUS serves more than 190 million people, providing services that range from primary care to highly complex procedures, with public university hospitals playing a prominent role. In addition to delivering care, these institutions play an essential role in the training of health professionals [^1^]. With Law No. 12,550, enacted on December 15, 2011, the Brazilian Hospital Services Company was created, currently known as HU Brasil, which is responsible for managing university hospitals [^2^].

According to HU Brasil, professionals working in these hospitals are categorized into three areas: medical (e.g., cardiologist, psychiatrist, pediatrician), health care (e.g., nurses, physical therapists, pharmacists), and administrative (e.g., administrative assistant, lawyer, administrator). These professionals are essential for hospital operations, contributing to health care delivery and the well-being of the Brazilian population.

During the COVID-19 pandemic, the challenges faced by health professionals worldwide led to an increased prevalence of symptoms of depression (29.4%) and anxiety (31.8%) [^3^]. This scenario was associated with greater exposure to psychosocial risks at work, such as workload overload, unhealthy environments, and risk of infection [^4^-^6^]. In addition, there was an increase in health-risk behaviors, such as excessive alcohol consumption and physical inactivity, which contribute to the worsening of these symptoms [^7^-^9^].

Symptoms of depression include depressed or irritable mood, reduced interest or pleasure, insomnia or hypersomnia, psychomotor agitation or retardation, fatigue or loss of energy, difficulty concentrating, recurrent thoughts of death, and suicidal ideation, with or without specific plans or suicide attempts. In turn, symptoms of anxiety include excessive worry, intense fear, restlessness, irritability, muscle tension, difficulty concentrating, fatigue, and avoidance behaviors [^10^].

In the treatment of symptoms of depression and anxiety, pharmacotherapy and psychotherapy are considered gold-standard interventions. Additionally, evidence indicates that modifying components of lifestyle medicine, such as reducing harmful habits and engaging in physical activity, is relevant in the context of mental health and contributes to the reduction of these symptoms [^11^-^13^].

Studies demonstrate that physical activity is effective in reducing symptoms of depression and anxiety through physiological adaptations associated with increased synthesis and release of neurotransmitters and neurotrophic factors, promoting processes such as neurogenesis, synaptogenesis, angiogenesis, and neuroplasticity [^13^].

In the context of occupational health, regular physical activity, whether in the workplace or structured outside of it, can prevent and reduce symptoms of depression and anxiety among health professionals and is recognized as an important strategy for health promotion and the prevention of physical and mental illnesses [^14^,^15^]. Furthermore, the literature highlights the importance of assessing mental health and associated risk factors in order to support the development of strategies to mitigate and monitor the long-term impacts of these symptoms at the individual, family, institutional, and social security levels [^16^-^20^].

However, there is a lack of evidence regarding levels of physical activity, the presence of symptoms of depression and anxiety, and their associations among professionals working in Brazilian university hospitals, particularly after the end of the Public Health Emergency of International Concern (PHEIC) related to COVID-19 [^21^]. In this context, a gap in the available scientific knowledge is identified, reinforcing the need for further investigations.

Thus, the aim of this study is to investigate the association between the level of physical activity and symptoms of depression and anxiety among professionals at a Brazilian university hospital after the end of the PHEIC related to COVID-19. The study hypothesis is that higher levels of physical activity are associated with lower symptoms of depression and anxiety among hospital professionals.

## METHODS

### STUDY DESIGN

This is an observational, cross-sectional, quantitative study approved by the Research Ethics Committee at Hospital Universitário Cassiano Antônio Moraes under CAAE: 74351123.1.0000.5071. The study followed the guidelines of the STrengthening the Reporting of OBservational Studies in Epidemiology statement [^22^], in its version for cross-sectional studies, and was conducted in accordance with the ethical principles of the World Medical Association [^23^].

Recruitment was conducted using nonprobabilistic convenience sampling. Data collection took place between December 2023 and May 2024 through an electronic form made available on the Google Forms platform. The access link was sent to participants via institutional email. Before completing the questionnaire, participants read and agreed to the informed consent form (ICF), which was provided in a dedicated section of the platform. Forms with duplicate responses, identified through participants’ email addresses, were excluded.

The questionnaire followed a sequential structure with linear and mandatory progression between pages. Skipping responses was not allowed, ensuring that all items were completed in a predefined order. The data collection platform allowed participants to pause and resume completion at a later time, with partial responses being saved. After submission, there was no pre-submission review stage, nor were copies of responses sent to participants.

The instrument was structured into five sections: Part I - Personal information (age, sex, weight, height, body mass index [BMI], and area of practice/job specialty, categorized as: i) medical: higher education/ specialist roles, such as angiologist, anesthesiologist, and nephrologist; ii) health care: technical and higher education/specialist roles, such as nursing technician, nurse, physical education professional, and psychologist; and iii) administrative: technical and higher education/specialist roles, such as administrative assistant, accounting technician, and administrative analyst); Part II - Self-reported health factors (presence of chronic diseases, use of continuous medication, alcohol consumption, and smoking); Part III - Symptoms of depression; Part IV - Symptoms of anxiety; and Part V - Level of habitual physical activity. The estimated time to complete the questionnaire was 15 to 20 minutes.

### PARTICIPANTS

The study included professionals working at a university hospital in the state of Espírito Santo, belonging to the three areas of practice (medical, health care, and administrative). Inclusion criteria were being employed at the hospital and voluntarily agreeing to participate in the study by signing the ICF. Professionals who did not agree to participate or did not complete the questionnaire were excluded.

### VARIABLES

#### Part I - Personal information

The first section of the questionnaire consisted of six items: age, sex, weight, height, BMI, and area of practice/ job specialty.

#### Part II - Health-related factors

In this section, participants reported self-perceived health factors, including the presence of chronic diseases, use of continuous medication, alcohol consumption, and smoking.

#### Part III - Symptoms of depression

Depressive symptoms and depression severity were assessed using the Beck Depression Inventory [^24^], which consists of 21 items, with a total score ranging from 0 to 63. The instrument evaluates cognitive, emotional, motivational, and somatic symptoms of depression. Scores were classified as follows: i) minimal or no depression (09), ii) mild to moderate depression (10-18), iii) moderate to severe depression (19-29), and iv) severe depression (30-63).

#### Part IV - Symptoms of anxiety

Symptoms of anxiety (trait anxiety) were assessed using the State-Trait Anxiety Inventory, translated and adapted into Portuguese [^25^]. The Trait Anxiety Scale was used, consisting of 20 statements related to individual personality characteristics. Total scores range from 20 to 80 and were classified as follows: i) low anxiety (20-40), ii) moderate anxiety (41-60), and iii) high anxiety (61-80).

#### Part V - Level of habitual physical activity

The level of habitual physical activity was assessed using the modified Baecke questionnaire [^26^]. The instrument includes three domains: i) activities of daily living, ii) sports activities, and iii) leisure activities. The activities of daily living domain consists of 10 closedended questions. The sports and leisure domains were assessed using open-ended questions, including type of activity, intensity, weekly frequency, and annual duration. Participants were classified based on the total score as: i) sedentary (< 9), ii) active (9-16), and iii) very active (> 16).

### ETHICAL ASPECTS

The ICF was obtained digitally through a specific section of the form, ensuring participants’ autonomy regarding study participation. Personal identifying data, such as names, were not collected or disclosed at any stage of the study. All information was handled with strict confidentiality, in accordance with ethical guidelines for research involving human subjects.

### STATISTICAL ANALYSIS

Data were collected using the Google Forms platform and exported to Microsoft Excel for preliminary processing. Statistical analyses were performed using Jamovi (version 2.5.7) and IBM SPSS Statistics for Windows, version 26 (IBM Corp., Armonk, N.Y., USA).

Data were presented using descriptive statistics, including measures of central tendency (median), measures of dispersion (interquartile range), and absolute and relative frequencies. Data normality was assessed using the Shapiro-Wilk test.

For comparisons between areas of practice, Fisher’s exact test and Yates’ continuity correction were used for qualitative variables, and the Kruskal-Wallis test was used for quantitative variables. For the Kruskal-Wallis test, Bonferroni post hoc analysis was applied, and effect size was estimated using epsilon squared (ɛ^2^).

To assess the association between independent variables (physical activity, alcohol consumption, and presence of chronic diseases) and psychiatric outcomes (symptoms of depression and anxiety), generalized linear models with normal distribution were used, selected based on the lowest Akaike Information Criterion. Both unadjusted (univariate analysis) and adjusted models (multivariate analysis) were tested, adjusting for sex, age, and BMI.

Effects were expressed using beta coefficients (β) and their respective 95%CIs. A significance level of p < 0.05 was adopted.

## RESULTS

A total of 199 hospital professionals (13.8% of the total) were included in the study, distributed across three areas of practice: medical (n = 19; 9.5%), health care (n = 137; 68.8%), and administrative (n = 43; 21.6%).

Table 1 presents the characterization of participants regarding personal information, health-related factors, level of physical activity, and scores of depression and anxiety symptoms. There was a predominance of females (n = 155; 77.9%), health professionals (n = 137; 68.8%), individuals with BMI above the normal range (n = 130; 65.3%), presence of self-reported chronic diseases (n = 119; 59.8%), use of continuous medication (n = 116; 58.6%), and sedentary behavior (n = 138; 69.3%).

**Table 1:** Absolute and relative frequency of sociodemographic characteristics, health factors, symptoms of depression and anxiety, and level of physical activity in professionals at a university hospital

Variable	n	%
Sex Male	44	22.1
Female	155	77.9
Body mass index Normal	69	34.7
Overweight	130	65.3
Area of practice Administrative	43	21.6
Health care	137	68.8
Medical	19	9.5
Chronic disease No	80	40.2
Yes	119	59.8
Medication use No	82	41.4
Yes	116	58.6
Alcohol consumption No	107	53.8
Yes	92	46.2
Smoking No	178	96.7
Yes	6	3.3
Depression Normal	111	55.8
Mild to moderate	54	27.1
Moderate to severe	24	12.1
Severe	10	5.0
Anxiety Low	91	45.7
Moderate	96	48.2
High	12	6.0
Physical activity Sedentary	138	69.3
Active	43	21.6
Very active	18	9.0

For descriptive purposes, 44.2% of participants of presented symptoms depression and 54.2% symptoms of anxiety.

In the descriptive analysis, hospital professionals were classified according to the severity of symptoms of depression and anxiety. Regarding depression, 27.1% presented mild to moderate symptoms, 12.1% moderate to severe symptoms, and 5.0% severe symptoms. Regarding anxiety, 48.2% of professionals presented moderate levels and 6.0% high levels.

Table 2 presents measures of central tendency and dispersion for age, body weight, height, BMI, and scores of depression, anxiety, and physical activity according to area of practice. Statistically significant differences between areas were observed only for age (p = 0.020) and body weight (p = 0.023).

**Table 2:** Measures of central tendency and dispersion of variables according to area of professional practice

		Area of practice		p-value	ɛ2
	Medical Median (IQR)	Health care Median (IQR)	Administrative Median (IQR)		
Age 40.00 (8.00)		42.50 (10.00)	39.50 (8.00)	0.020^*^	0.042
Body weight 68.00 (18.50)		73.00 (20.70)	79.00 (27.70)	0.023^*^	0.037
Height 167.00 (12.00)		165.00 (12.00)	166.00 (15.50)	0.206	0.013
BMI 29.00 (7.90)		26.60 (6.55)	24.10 (5.60)	0.086	0.020
Depression 8.00 (6.50)		9.00 (12.00)	6.50 (10.00)	0.239	0.013
Anxiety 41.00 (10.50)		42.00 (17.50)	39.00 (15.70)	0.269	0.014
Physical activity score 7.00 (7.00)		4.53 (7.50)	4.20 (7.60)	0.296	0.012

* Statistically significant difference (p < 0.05).

BMI: body mass index; IQR: interquartile range.

Table 3 presents the results of the simple linear regression. The presence of chronic disease was positively associated with depression scores (β = 0.39; p = 0.013; 95%CI: 0.08-0.71) and anxiety scores (β = 0.12; p = 0.030; 95%CI: 0.04-0.21). Alcohol consumption showed a positive association with symptoms of anxiety (β = 0.09; p = 0.006; 95%CI: 0.02-0.15).

**Table 3:** Simple linear regression analysis of factors associated with symptoms of depression and anxiety in professionals at a university hospital

Variables	Depression		Anxiety	
	β (95%CI)	p value	β (95%CI)	p-value
Chronic disease (yes vs. no)	0.39 (0.08-0.71)	0.013^*^	0.12 (0.04-0.21)	0.030^*^
Alcohol consumption (yes vs. no)	-	-	0.09 (0.02-0.15)	0.006^*^
Physical activity (yes vs. no)		0.001^*^		0.017^*^
Very active	-0.43 (-0.91 to 0.05)	-	-0.07 (-0.17 to 0.02)	-
Active	-0.41 (-0.64 to -0.18)	-	-0.09 (-0.16 to 0.02)	-
Sedentary	1	-	1	-

* Statistically significant difference (p < 0.05).

Physical activity showed a significant inverse association with depression and anxiety scores. For depression, negative associations were observed for active individuals (β = -0.41; p = 0.001; 95%CI: -0.64 to -0.18) and very active individuals (β = -0.43; p = 0.001; 95%CI: -0.91 to -0.05), compared with sedentary individuals. For anxiety, inverse associations were also observed for active individuals (β = -0.09; p = 0.017; 95%CI: -0.16 to -0.02) and very active individuals (β = -0.07; p = 0.017; 95%CI: -0.17 to -0.02).

Table 4 presents the results of the adjusted multivariate linear regression, stratified by area of practice. Among professionals in the medical field, age was inversely associated with depression scores (β = -0.41; p < 0.001; 95%CI: -0.61 to -0.21) and anxiety scores (β = -0.39; p = 0.040; 95%CI: -0.77 to -0.01).

**Table 4:** Adjusted multivariate linear regression analysis, stratified by role (medical, health care, and administrative), of factors associated with symptoms of depression and anxiety among professionals at a university hospital

		Depression				
	**Medical area**		**Health care area**		**Administrative area**	
**Variable**	**β (95%CI)**	**p-value**	**β (95%CI)**	**p-value**	**β (95%CI)**	**p-value**
Age	-0.41 (-0.61 to -0.21)	< 0.001^*^	0.08 (-0.10 to 0.27)	0.371	-0.16 (-0.51 to 0.18)	0.362
Sex	0.91 (-5.59 to 7.42)	0.783	-1.94 (-5.18 to -1.29)	0.240	-2.81 (-8.54 to 2.92)	0.337
BMI	-0.25 (-0.80 to 0.29)	0.366	0.18 (-0.11 to 0.48)	0.231	0.38 (-0.26 to 1.03)	0.246
Chronic disease	-5.01 (-8.64 to -1.38)	0.007^*^	4.41 (1.27-7.56)	0.006^*^	2.97 (-3.34 to 9.28)	0.356
Physical activity						
Very active	-12.17 (-16.58 to-7.76)	< 0.001^*^	-3.95 (-6.41 to -1.49)	0.002^*^	1.56 (-11.5 to 14.62)	0.815
Active	-8.22 (-12.84 to -3.60)	< 0.001^*^	-4.30 (-6.88 to -1.71)	0.001^*^	-1.33 (-6.69 to 4.03)	0.626
Sedentary	1	-	1	-	1	-
			**Anxiety**			
	**Medical area**		**Health care area**		**Administrative area**	
**Variable**	**P (95%CI)**	**p-value**	**P (95%CI)**	**p-value**	**P (95%CI)**	**p-value**
Age	-0.39 (-0.77 to-0.01)	0.040^*^	-0.06 (-0.27 to 0.14)	0.556	-0.19 (-0.61 to 0.23)	0.385
Sex	2.17 (-8.11 to 12.45)	0.679	-2.79 (-7.27 to 1.69)	0.222	-1.94 (-8.47 to -4.57)	0.559
BMI	-0.80 (-1.37 to -0.24)	0.005^*^	0.25 (-0.13 to 0.65)	0.202	0.61 (-0.29 to 1.52)	0.180
Chronic disease	-10.18 (-15.66 to -4.69)	< 0.001^*^	7.14 (3.30-10.98)	< 0.001^*^	2.44 (-8.89 to 13.77)	0.673
Physical activity						
Very active	-20.05 (-27.12 to -12.97)	< 0.001^*^	-2.01 (-5.99 to 1.97)	0.32	1.39 (-10.02 to 12.82)	0.810
Active	-7.20 (-15.14 to 0.74)	0.076	-5.55 (-8.69 to -241)	0.001^*^	-1.47 (-7.79 to 4.85)	0.648
Sedentary	**1**	-	1	-	1	-

* Statistically significant difference (p < 0.05).

Models adjusted for sex, age, and BMI.

BMI = body mass index.

The presence of chronic disease was associated with lower depression scores (β = -5.01; p = 0.007; 95%CI: -8.64 to -1.38) and lower anxiety scores (β = -10.18; p < 0.001; 95%CI: -15.66 to -4.69).

Physical activity level showed an inverse association with depression and anxiety scores. For depression, active (β = -8.22; p < 0.001; 95%CI: -12.84 to -3.60) and very active individuals (β = -12.17; p < 0.001; 95%CI: -16.58 to -7.76) had lower scores compared with sedentary individuals. For anxiety, a significant inverse association was observed for active and/or very active individuals (β = -20.05; p < 0.001; 95%CI: -27.12 to -12.97), compared with the sedentary group.

Among professionals in the health care field, the presence of chronic disease was associated with higher depression scores (β = 4.41; p = 0.006; 95%CI: 1.27-7.56) and higher anxiety scores (β = 7.14; p < 0.001; 95%CI: 3.30-10.98). Physical activity remained inversely associated with the scores. Significant reductions in depression scores were observed among active (β = -4.30; p = 0.001; 95%CI: -6.88 to -1.71) and very active individuals (β = -3.95; p = 0.002; 95%CI: -6.41 to -1.49), compared with sedentary individuals. For anxiety, physical activity was associated with reduced scores only among active individuals (β = -5.55; p = 0.001; 95%CI: -8.69 to -2.41).

In the administrative group, no statistically significant associations were observed between the independent variables evaluated and depression or anxiety scores after adjustment.

## DISCUSSION

The aim of this study was to investigate the association between the level of physical activity and symptoms of depression and anxiety among professionals at a Brazilian university hospital after the end of the PHEIC related to COVID-19. The results confirm the proposed hypothesis, showing a significant inverse association between the level of physical activity and scores of depression and anxiety assessed using standardized instruments.

Supporting these findings, Bernhard et al. [^16^], in a study conducted during the COVID-19 pandemic with health professionals, reported that individuals classified as active or irregularly active had lower severity of symptoms of depression and anxiety compared with sedentary individuals, suggesting a consistent association between physical activity and better mental health indicators. Similarly, Martín Del Campo et al. [^28^] observed that higher levels of moderate physical activity, assessed before the pandemic, were significantly associated with lower severity of symptoms of depression, anxiety, and stress at the peak of COVID-19 among professionals in a tertiary hospital, reinforcing this relationship in the occupational context.

Regarding the descriptive characterization of the sample, 44.2% and 54.2% of participants presented symptoms of depression and anxiety, respectively, according to the cutoff points of the scales used. These values are higher than those reported in the literature during the pandemic. Al Maqbali et al. [^3^], in a review including 72 meta-analyses and 2,308 primary studies, identified pooled prevalences of 29.4% for symptoms of depression and 31.8% for anxiety among health professionals during the pandemic period.

Thus, the findings indicate that the frequency of symptoms of depression and anxiety among professionals at a Brazilian university hospital in the post-pandemic period remained high when analyzed categorically. However, the inferential analyses in this study were conducted based on continuous scores, allowing for a more sensitive evaluation of the magnitude of the association between physical activity and symptom levels.

Regarding the level of physical activity, 69.3% of professionals were classified as sedentary, a proportion higher than that reported in previous studies. For example, Fond et al. [^29^], when analyzing 10,325 health professionals, found that 38.1% had insufficient levels of physical activity.

Additionally, the presence of chronic disease was associated with higher depression scores, which may be explained by the functional impact of the condition, persistent symptom burden, and stress related to ongoing disease management, factors known to increase vulnerability to depressive symptoms. Alcohol consumption was also associated with higher anxiety scores, a finding consistent with evidence indicating a bidirectional relationship in which alcohol use may act as a dysfunctional coping strategy while also contributing to the maintenance or worsening of anxiety symptoms [^30^].

This study has relevant limitations. Due to its cross-sectional design, it is not possible to establish temporality between physical activity and mental health outcomes, which limits causal inferences. Furthermore, the nonprobabilistic sample, composed of professionals from a single hospital, may have introduced selection bias and limits the generalizability of the findings.

The stratified analysis by area of practice should be interpreted with caution, especially in the medical group (n = 19), whose small sample size may compromise the stability of estimates and statistical power. The inverse associations observed between age, BMI, chronic disease, and symptoms in this group, although counterintuitive, likely reflect imprecision due to the small number of participants, reinforcing the need for cautious interpretation.

Finally, the use of a digital questionnaire with selfreported measures may have introduced information bias, particularly related to item interpretation and response accuracy. Despite these limitations, the findings provide relevant evidence on the relationship between physical activity and mental health among health professionals and may support health promotion strategies and guide future studies with longitudinal designs and more robust samples.

## CONCLUSIONS

An inverse association was observed between the level of physical activity and symptoms of depression and anxiety among professionals at a Brazilian university hospital, such that higher levels of physical activity were significantly associated with lower scores of these symptoms. Considering these findings and the limitations inherent to the study design and sample scope, further studies are recommended in other university hospitals with larger samples to deepen the understanding of these associations and their implications for the development of occupational health promotion strategies.

## Data Availability

The data supporting the findings of this study are available within the article.

## Statement on the use of the language models

The authors declare that they used the ChatGPT language model to assist with writing, translation, revision, and synthesis of the manuscript, ensuring that they reviewed all the content of the material after the revisions.
